# Cadences of the collective: conspecific stimulation patterns interact with endogenous rhythms to cue socially mediated response shifts

**DOI:** 10.1242/jeb.250982

**Published:** 2026-01-06

**Authors:** Luke C. Larter, Colby W. Cushing, Michael J. Ryan

**Affiliations:** ^1^ Institute at Brown for the Environment and Society, Brown University, 85 Waterman St, Providence, RI 02912, USA; ^2^ Department of Ecology, Evolution, and Organismal Biology, Brown University, Providence, RI 02912, USA; ^3^ Smithsonian Tropical Research Institute, Apartado 0843-03092, Balboa, Republic of Panama; ^4^ Department of Integrative Biology, University of Texas at Austin, 2415 Speedway, Austin, TX 78712, USA; ^5^ Applied Research Laboratories, The University of Texas at Austin, Austin, TX 78758, USA

**Keywords:** Collective behavior, Chorusing, Sensory scenes, Sexual selection, Automated playback

## Abstract

Many animals form behavioral collectives, and optimal interaction strategies often differ across social contexts. Sensory scenes generated by many interacting conspecifics are complex. Thus, maintaining socially calibrated responses requires individuals to distill key features from conspecific scenes to guide continued adjustments to social fluctuations. Túngara frogs produce mating calls in choruses varying in size, and interaction patterns differ across social environments; rivals alternate their calls in smaller choruses, but increasingly overlap one another's calls in a stereotyped fashion as chorus size increases. We used automated playback to investigate the cues guiding this socially mediated shift in interaction modes. We played conspecific stimulus calls to males at various delays relative to their own calls, preceded by various acoustic motifs that mimicked conspecific stimulation patterns males will hear in different social environments. Males almost never overlapped isolated stimulus calls at any delays. However, their probabilities of overlapping stimulus calls increased markedly when stimulus calls were preceded by motifs characteristic of larger choruses, i.e. those exhibiting intense conspecific stimulation patterns. Furthermore, the escalatory effects of motifs became increasingly pronounced as motif/stimulus combinations were played at later delays. Thus, interaction strategies are calibrated to current social dynamics each call cycle in response to a multifaceted cue that incorporates both the nature of conspecific stimulation experienced and how the timing of this stimulation interacts with endogenous responsiveness rhythms. Our results highlight that inactive phases within behavioral rhythms provide repeated opportunities to sample current social dynamics, allowing response patterns to be continually calibrated to social fluctuations in behavioral collectives.

## Teaser

**Summary:** Automated playback experiments reveal that túngara frogs alter each calling response based on conspecific stimulation patterns experienced prior to each call.

## INTRODUCTION

Many fitness-enhancing behaviors require successful interactions with conspecifics (Alexander, 1974). This selects for sensory tuning and cognitive abilities that allow individuals to glean salient information from conspecific cues and signals to guide adaptive responses. Conspecific signals, and potential cues, are typically complex and vary along many dimensions. However, not all features may be equally valuable for guiding appropriate responses, or properties of perceptual systems may constrain receivers' abilities to use potentially informative variation along all dimensions. Consequently, perceptual systems simplify incoming streams of sensory information by prioritizing processing of only the most salient features of conspecific cues and signals (Capranica, 1978; Hein, 2022; Wilczynski and Ryan, 2010). For instance, schooling and swarming behaviors can be elicited from fish and locusts by simple moving shapes (Bleichman et al., 2023; Harpaz et al., 2021). Similarly, frogs respond to highly simplified synthetic versions of conspecific calls that retain only a few crucial features, such as species-specific note rates and general frequency patterns (Feng et al., 1990; Gerhardt, 1974; Wilczynski et al., 1995). That coarse approximations elicit appropriate social responses demonstrates that animals' perceptual systems distill the most salient patterns from conspecific signals and cues, then rely on these essential features for guiding adaptive responses (Tinbergen, 1951).

In addition to responding appropriately to conspecifics during one-on-one interactions, animals must effectively navigate diverse social environments consisting of varied numbers and identities of conspecifics. Optimal interaction strategies often differ by social context, which selects for socially mediated flexibility in response patterns. For instance, courting signalers increase signaling effort in more competitive signaling environments (Greenfield, 2015; Wells, 2007), combatants alter aggressive tactics based on audience composition (Matos and Schlupp, 2005), and collectively locomoting individuals maintain different nearest-neighbor distances in groups of different sizes (Harpaz et al., 2024 preprint; Ward et al., 2017). Sensory scenes generated in crowded social environments are invariably more complex than those generated by individual conspecifics (Bee and Micheyl, 2008; Hein, 2022; Williams et al., 2023). Thus, in addition to distilling salient features from individual cues and signals, these examples demonstrate that animals' perceptual systems can also distill informative features from broader conspecific scenes and use them to guide flexible responses.

Scenes composed of many interacting individuals contain features of the individuals within them, as well as emergent features and patterns arising as a result of interactions among these individuals. Therefore, establishing precisely which aspects of these dynamic and multifaceted scenes are salient for guiding behavior can be challenging (Harpaz et al., 2024 preprint; Williams et al., 2023). Identifying salient features is complicated further by the fact that endogenous behavioral rhythms can influence how individuals interpret and respond to conspecific stimulation. This means that social cues can include a temporal component; it may not simply be the properties of conspecific stimulation that matter, but also when this stimulation is perceived relative to endogenous rhythms. For instance, competitive signaling interactions in *Mecapoda* katydids involve callers delaying or advancing their upcoming call in response to a rival's call, and these temporal adjustments differ depending on when this rival's call is perceived relative to endogenous calling rhythms (Hartbauer et al., 2005; Sismondo, 1990). Similarly, juvenile zebrafish swimming dyadically alter the intervals between successive locomotion bursts as a function of the delay between their most recent burst and their partner's response burst, generating temporally coupled bursting dynamics (Amichay et al., 2024). Beyond one-on-one interactions, behavioral rhythms can also influence individuals' responses to emergent regularities in collective conspecific scenes. For instance, marching locusts and schooling zebrafish alternate between phases of rapid and slowed locomotion, and are more visually responsive to the predominant movement directions and velocities of group members during slowed phases (Aidan et al., 2024; Ariel et al., 2014; Harpaz et al., 2017). Thus, in many collective contexts, salient cues guiding socially mediated flexibility are complex and multi-dimensional, with responses being guided by how emergent conspecific stimulation patterns interact with individuals' endogenous responsiveness rhythms (Larter and Ryan, 2025).

Acoustic chorusing is a collective behavioral context in which the rhythmicity of behavior, and socially mediated flexibility, have been extensively studied; many playback experiments have probed the temporal coupling functions facilitating dyadic calling interactions (Greenfield, 1994; Klump and Gerhardt, 1992; Larter and Ryan, 2025), and many have investigated how callers alter their calling strategies across different social environments (Greenfield, 2015; Wells, 2007). However, no studies have experimentally investigated the role that interactions between emergent regularities in conspecific scenes and individuals' endogenous calling rhythms play in guiding flexible interaction strategies. Here, we utilized an automated playback paradigm to investigate whether such an interaction cues socially mediated shifts in response modes in túngara frogs. This species shows two largely discrete response types; a male can call in alternation with a rival's call without overlapping it, or can overlap it in a stereotyped way (Larter and Ryan, 2024a; described in Materials and Methods). Prevalences of these different response types strongly correlate with chorus size, with alternation predominating in smaller choruses and overlap becoming increasingly prevalent as choruses grow beyond three males. Furthermore, observations hint that overlap is driven by an interaction between endogenous calling rhythms and broader conspecific stimulation patterns (Larter and Ryan, 2024c).

To explore this experimentally, we played conspecific stimulus calls to males at various delays relative to their own calls, and preceded these stimulus calls with a variety of acoustic motifs that mimicked conspecific stimulation patterns males will hear in different social environments. We hypothesized that the probability that a male overlapped the stimulus call would be influenced by an interaction between the motif preceding the stimulus call and the delay at which he encountered it relative to his most recent call. We predicted that: (i) stimulus calls preceded by motifs typical of more crowded choruses would have higher probabilities of being overlapped, (ii) stimulus calls encountered at later delays throughout males' call cycles would have higher probabilities of being overlapped (Larter and Ryan, 2024c), and (iii) that these factors would interact, such that overlap probability would be increased synergistically when stimulus calls were both preceded by crowded chorus motifs and encountered at later delays. Furthermore, males exhibit socially dependent flexibility in call elaboration patterns (Bernal et al., 2007; Larter and Ryan, 2024a; Larter et al., 2023) and response latencies to offsets of rivals' calls when alternating (Larter and Ryan, 2024a), and we predicted that analogous synergistic effects would also underpin flexibility in these aspects of calling behavior.

## MATERIALS AND METHODS

### Túngara frogs

**Fig. 1.**
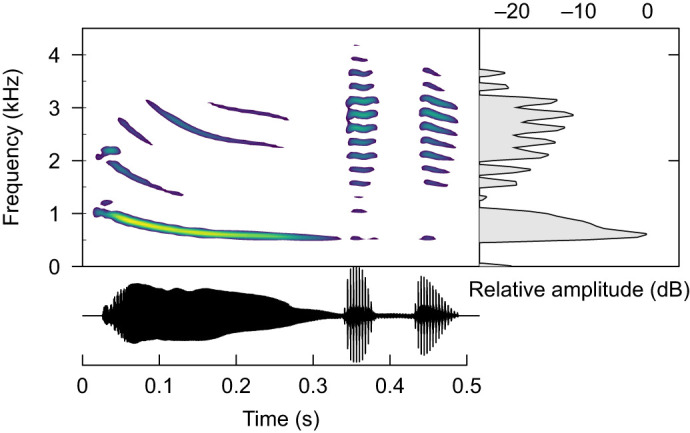
**A spectrogram (top), waveform (bottom) and power spectrum (right) of a complex túngara frog call.** Data were processed with the ‘seewave’ R package, window length 1024, overlap 90%. Brighter colors denote higher intensities. This call begins with a continuous ‘whine’ note and ends with two ‘chuck’ notes.

Túngara frog, *Physalaemus* (=*Engystomops*) *pustulosus* (Cope 1864), males form choruses in shallow puddles and pools. Choruses can be dense, and density can vary within the same area on the same night (Bernal et al., 2007). This species' calls begin with a ‘whine’, a descending frequency sweep, to which can be added one or more broadband ‘chuck’ notes (Fig. 1; Ryan, 1985). Calls including chucks (‘complex calls’) are 5-fold more attractive to females than whines alone (‘simple calls’) (Ryan et al., 2019). Median call periods (time elapsing between the onset of one call and the next for a given caller) are ∼1.7 s for chorusing males (Larter and Ryan, 2024a).

Calling interactions among rivals are mediated by a ‘gap-detection’ strategy. Here, calls are triggered when males experience an abrupt reduction in inhibition by acoustic stimulation. This is because such reductions in inhibition are indicative of the onset of a low-interference ‘gap’ within ongoing chorus noise into which an impending call can be inserted (Larter and Ryan, 2024b, 2025). However, interaction patterns differ by chorus size. In choruses of three or fewer males, rivals alternate their calls without overlapping. Yet, above this threshold, call overlap among rivals becomes increasingly prevalent. This overlap is highly stereotyped, with the whine onset of the overlapping following call beginning just before the chucks of the overlapped leading call (Larter and Ryan, 2024a). For following males, overlapping rivals in this way induces lower attractiveness costs than any other potential form of overlap while also reducing the attractiveness of the overlapped leading call. Thus, this is a beneficial interaction pattern in larger choruses where overlap becomes unavoidable (Larter and Ryan, 2024a).

Furthermore, both alternation and stereotyped call overlap seem to be facilitated by the same gap-detection mechanism. Amplitude and frequency decrease concurrently throughout whines, and male sensory tuning translates this concurrent decrease into a moderately steep release from inhibition shortly after whine onsets, which can trigger calls under certain circumstances (Larter and Ryan, 2024b; illustrated in Larter and Ryan, 2025). Thus, alternation in smaller choruses occurs because the stark release from inhibition accompanying the offsets of rivals' calls to silence is a highly salient call trigger. Conversely, stereotyped overlap in larger choruses arises as the moderately steep release from inhibition occurring just after whine onsets becomes an increasingly salient call trigger as choruses grow larger (Larter and Ryan, 2024a,b). Thus, the salience of different call triggers varies across social environments, driving varied interaction patterns. Furthermore, chorusing males were more likely to overlap rivals' calls encountered later throughout their call cycles, suggesting the salience of this call trigger is also mediated by males' endogenous calling rhythms (Larter and Ryan, 2024c).

Other aspects of male calling behavior also correlate with chorus size. In larger choruses, males increase call elaboration by appending more chucks to calls (Bernal et al., 2007; Larter and Ryan, 2024a) and increasing chuck amplitude (Halfwek et al., 2015), and decrease response latencies to rivals' call offsets when alternating with them (Larter and Ryan, 2024a).

### Playback experiments

#### Experimental subjects

During July and August 2024, we collected males as members of amplectant pairs from urban breeding sites around Gamboa, Panama, near the Smithsonian Tropical Research Institute grounds. We separated males from their associated females for trials and, following trials, weighed males (g), and gave them a unique toe clip to avoid retesting. We then reunited males with their females and returned pairs to their capture locations later that same night. Morphological and environmental data for 3 of 39 males were lost, so we assigned these males median masses and water temperatures.

All research was permitted by the Government of Panama (ARB-130-2023), approved by STRI-ACUC (SI-24020-1) and UT Austin IACUC (AUP-2022-00012), and followed the Guidelines for Use of Live Amphibians and Reptiles in Field and Laboratory Research (Beaupre et al., 2004).

#### Stimuli

**Fig. 2.**
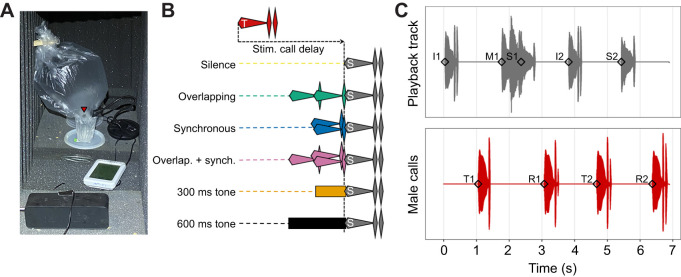
**Illustrations of automated playback trials.** (A) A male (red arrow) in our automated playback setup. The microphone registered his call onsets, which triggered stimuli to be played from the speaker in the foreground. (B) Illustrations of acoustic motifs that preceded stimulus calls. Red ‘T’ call represents the male's trigger call, and gray ‘S’ calls represent stimulus calls that could be played back at an array of stimulus delays, and could be preceded by any of these motifs. (C) An example of a male (lower trace) interacting with automated playback (upper trace). In the playback track, I1 and I2 denote onsets of ‘interim calls’, S1 and S2 denote onsets of ‘stimulus calls’, and M1 denotes the onset of the ‘motif’ preceding S1 (here, overlapping+synchronous). S2 here is preceded by silence. In the male response track, T1 and T2 denote onsets of ‘trigger calls’ that initiate stimulus call playback, and R1 and R2 denote onsets of ‘response calls’ to these stimulus calls. Key variables of interest are visible here: (i) ‘stimulus delay’: the time elapsing between the onset of a trigger call and the onset of the consequent stimulus call [e.g. S1–T1 (not M1–T1)]; our temporal predictor variable of interest. (ii) ‘Response delay’: time elapsing between the onset of a stimulus call and the onset of the subsequent male response call (e.g. R1–S1); a temporal response variable of interest. (iii) ‘Response period’: time elapsing between the onset of a male's trigger call and the onset of his subsequent response call following stimulus presentation (e.g. R1–T1); another temporal response variable of interest, equivalent to ‘stimulus delay’+‘response delay’. (iv) ‘Call elaboration’: the summed amplitude, relative to the peak amplitude of the associated whine, of all chuck notes appended to a call. Increasing call elaboration is visible throughout the lower trace.

For stimulus calls, we chose three calls from a previous recording, each made by a different male as they called together as members of the same six-male chorus (Larter and Ryan, 2024c). These males differed markedly in their probabilities of being overlapped by their rivals, as ascertained by extracting males' random intercepts from the generalized linear mixed-effects model (GLMM) predicting overlap probability in that paper. Prior to doing so, we removed all pertinent fixed effects to concentrate all inter-male variation in this random intercept. ‘Stimulus call IDs’ denote these relative differences among stimulus calls in previously observed overlap probabilities (OP): ‘low OP’ [*P*(overlapped)=0.18], ‘intermediate OP’ [*P*(overlapped)=0.52], and ‘high OP’ [*P*(overlapped)=0.84]. Durations of these calls correlated with overlap probabilities (low OP 0.428 s<intermediate OP 0.484 s<high OP 0.519 s). However, looking more closely at overlap patterns revealed that differences in overlap probability were not driven by differences in duration per se, which we discuss in the Results. We also chose a fourth call, by a male from a different chorus that also had a low probability of being overlapped by his rivals [*P*(overlapped)=0.12]. We refer to this as the ‘interim call’, and responses to this call were not tested. Rather, the interim call was played to males at a fixed delay in between each stimulus call presentation (discussed below), and we used interim calls to construct the acoustic motifs that preceded stimulus calls (Fig. 2; discussed below).

We presented stimulus calls to males at various delays relative to their own calls, either in isolation (preceded by silence) or preceded by acoustic motifs composed of combinations of overlapping calls, synchronous calls or tones (Fig. 2). Overlapping and synchronous call motifs mimicked real interactions observed in larger túngara frog choruses (Larter and Ryan, 2024a,c). Motifs were constructed using our interim call with its second chuck removed. This call previously had a low probability of being overlapped, suggesting males would be stimulated by our motifs but would seldom overlap them, thereby focusing their responses on the stimulus calls of interest that followed motifs. Additionally, to disentangle the effects of more specific features of conspecific stimulation versus merely inhibition by intense acoustic stimulation generically, we included motifs consisting of 1000 Hz tones, which strongly inhibit calling (Larter and Ryan, 2024b). Thus, stimulus calls could be preceded by: (i) silence (i.e. isolated stimulus calls), (ii) two overlapping calls (610 ms in duration), (iii) two synchronous calls (370 ms), (iv) a combination of three calls exhibiting both overlap and synchrony (denoted ‘overlapping+synchronous’ to avoid confusion; 610 ms), (v) a 610 ms 1000 Hz tone, or (vi) a 310 ms 1000 Hz tone (Fig. 1). The last 10 ms of motifs overlapped with the first 10 ms of stimulus calls, to generate smoother transitions free from low-amplitude dips, meaning motifs extended up to 600 ms before stimulus call onsets. Motifs are visualized in Fig. S1.

Stimulus calls preceded by silence were played at eight ‘stimulus delays’ relative to the onset of the male's trigger call (his call that triggered playback of the stimulus call): 0.45 s (the duration of a typical call: Ryan and Rand, 2003), 0.6 s, 0.75 s, 0.9 s, 1.05 s, 1.2 s, 1.35 s and 1.5 s. Typical call periods are 1.7 s. However, males sometimes called spontaneously just prior to onsets of stimulus calls presented at later delays, which also gave us responses to stimulus calls that overlapped trigger calls. However, these responses were biased towards males with the fastest intrinsic call rates (discussed in the Results). Conversely, as acoustic motifs extended up to 0.6 s before stimulus call onsets, we could only play stimulus calls preceded by them at later stimulus delays: 1.05 s, 1.2 s, 1.35 s and 1.5 s. To clarify, stimulus delays denote the delay between the onset of the subject's trigger call and the onset of the consequent stimulus call, not the onset of the motif preceding this stimulus call (see Fig. 2).

#### Automated playback trials

Trials took place in a darkened room. During trials, a male called while floating on the surface of water contained in a 6.5 cm-diameter cup within an acoustically transparent enclosure (Fig. 2). Water temperature ranged from 26.4 to 28.6°C. A JOUNIVO USB microphone registered the male's call onsets to facilitate interactions with our automated playback program. Playback stimuli were played from an ANKER SoundCore speaker positioned 38 cm from the center of the male's cup. We calibrated this speaker such that peak amplitudes of whines of stimulus and interim calls, and 1000 Hz tones, registered at 82 dB sound pressure level (SPL) (re. 20 µPa) (the amplitude of a typical call from ∼1.3 m away) at the center of the cup as measured by a PCE-322A SPL meter (C-weighted, fast). This setup was surrounded by acoustically insulating foam.

The microphone and speaker were connected to a laptop running our automated playback program via a Jupyter (v7.0.8) notebook coded in Python (v3.11.7), that utilized functionalities from the PyAudio package (v0.2.14). This allowed us to play stimulus calls to males at our desired array of stimulus delays. A brief outline of playback trials is as follows. (1) At the start of trials, males were stimulated to call via 1 min of playback of interim calls. (2) The onset of the male's call in response to the final interim call in this sequence then triggered playback of the first stimulus call in the trial. (3) This stimulus call was played to the male at the desired stimulus delay, and the onset of his call in response to this stimulus call then triggered an interim call to be played at a fixed delay of 0.75 s. (4)  The onset of the male's call in response to this interim call then triggered the next stimulus call in the sequence. Then, back to step 3.

Steps 3 and 4 then alternated, with stimulus and interim calls presented alternately for remaining stimuli (Fig. 2). This ensured a ‘palette-cleansing’ alternation interaction with an interim call between each stimulus call presentation, to reduce carry-over effects. All stimulus call/stimulus delay/motif combinations were played to each male in a unique randomized order within a single trial, with each combination appearing 5 times (490 total stimulus presentations in a full trial). Full trials lasted ∼35 min, but we ended trials early if males ceased calling for several minutes. Time stamps recorded by our automated playback program for trigger, response and stimulus call onsets were not sufficiently precise or consistent for analysis (+-50 ms deviation at most). Thus, we also recorded the subject and playback speaker via separate Synco LavS6R tie-clip microphones onto a Zoom F6 recorder. We extracted precise time stamps from these recordings using functions from the ‘Librosa’ Python package (McFee et al., 2015) and only used these time stamps and delays calculated from them in our analysis.

### Data analysis

#### Response delay and response period curves

Phase response curves are commonly used to investigate the temporal responses of calling males to rivals' calls encountered at different points throughout their call cycles (Greenfield, 1994; Larter and Ryan, 2025). We constructed analogous curves to characterize male temporal responses to stimulus calls across our range of stimulus delays. However, rather than normalizing stimulus and response delays to phase angles, we modeled them in absolute time and accounted for inter-male variation using random effects.

We modeled temporal responses to stimulus calls presented at different stimulus delays (Response_Delay_GAMM: response delay∼stimulus delay), and the consequences of these responses for male calling rhythms (Response_Period_GAMM: response period∼stimulus delay) using generalized additive mixed-effects models (GAMM) with inverse-gaussian distributions (‘mgcv’ R package: Wood, 2001). Here, we only included responses to stimulus calls preceded by silence, and excluded responses where males overlapped stimulus calls rather than alternating with them [only 150/4399 (3.5%) of responses]. This yielded *n*=4249. For Response_Delay_GAMM, we included response delay as the response variable and, for Response_Period_GAMM, we included response period as the response variable. Otherwise, these models were identical. We included stimulus delay as a predictor variable and modeled this relationship with a non-linear smooth containing 10 knots. We also included a random intercept and random smooths for individual males, to capture inter-male variation. Additionally, we included linear covariates expected to influence responses; (i) the onset time of response calls within trials (to control for potential fatigue or habituation effects), (ii) the call elaboration score of the trigger call initiating each playback (standardized within males; to control for within-male variability in general arousal levels throughout trials), (iii) stimulus call ID as an unordered factor (low OP, intermediate OP and high OP), (iv) male mass (in grams) and (v) water temperature at trial beginning (°C). We included random slopes for most linear variables, except for mass and water temperature, for which males only experienced a single value. Throughout our analysis, we checked model assumptions using the ‘DHARMa’ R package (https://CRAN.R-project.org/package=DHARMa); all models and checks can be rerun using the associated R code.

#### Effects of preceding motifs on responses to stimulus calls

We used mixed effects models (‘lme4’ R package: https://CRAN.R-project.org/package=lme4) to investigate how male responses to stimulus calls were influenced by interactions between stimulus call ID, the motifs preceding them and the stimulus delays at which they were played. To minimize risks of type 1 errors (falsely significant results), we did not simplify the fixed-effect structure of models (Schielzeth and Forstmeier, 2009), and we fitted the maximal random-effects structures supported by the data (Barr et al., 2013). We included random intercepts for subject identity nested within testing night, and initially included correlated random slopes for all predictor variables (except mass and water temperature for which each subject only experienced a single value). However, when correlated random slopes for certain variables were not supported, we removed correlation terms, then removed these random slopes entirely if they remained unsupported. We standardized all continuous predictor variables {[*x*−mean(*x*)]/s.d.(*x*)} prior to analysis, with trigger call elaboration score being standardized within males to account for the large degree of intermale variation in typical elaboration levels (Larter and Ryan, 2024a).

##### Overlap_GLMM

To investigate the factors responsible for inducing overlapping responses, we built a mixed effects logistic regression model with whether the response call onset occurred anywhere during the stimulus call or not as the response variable (*n*=12,153). As predictor variables, we included stimulus delay, motif preceding the stimulus call and stimulus call ID (low OP, intermediate OP, high OP). As we anticipated these factors would interact, we included all two-way interactions between these variables. We also included the same control covariates as in our GAMMs: the onset time of male response calls, trigger call elaboration score, male mass and water temperature.

##### Response_Delay_LMM

To investigate the factors influencing response delays, we built a log-linear mixed effects model with log(response delay) as the response variable. Here, we only included the subset of responses that did not overlap stimulus calls, i.e. alternated with them, allowing us to investigate response delays relative to an identifiable call-trigger (stimulus call offsets) (*n*=8863). We included the same predictor variables and interactions as in Overlap_GLMM.

##### Within_Male_Call_Elaboration_LMM

Typical call elaboration levels vary among males (Larter and Ryan, 2024a), and males adjust call elaboration levels across chorus densities (Bernal et al., 2007). To investigate how our factors of interest influenced short-term within-male changes in call elaboration, we built a linear mixed effects model with response call elaboration score as the response variable (*n*=12,153) and trigger call elaboration score as a control covariate. Importantly, both trigger and response call elaboration scores were standardized using their collective mean and standard deviation. Thus, this model reveals to what degree different playback stimuli combinations induced an increase or decrease in response call elaboration scores relative to the trigger call immediately prior to them, adjusted to account for differences in males' typical call elaboration levels. We included the same predictor variables and interactions as in Overlap_GLMM except that, as we were modeling within-male call elaboration patterns, we did not include mass and water temperature as each male only experienced a single value and their effects would be subsumed by our procedure of standardizing response call elaboration scores within males.

##### Between_Male_Call_Elaboration_LMM

To investigate how phenotypic (body mass) and environmental (water temperature) factors contribute to between-male differences in call elaboration scores, we built a log-linear mixed effects model with log(unstandardized response call elaboration score) as the response variable. Log-linear models cannot handle zeroes, so we removed 12 calls with response call elaboration scores of 0 (*n*=12,141). Here, we included the same predictor variables as Overlap_GLMM, except that we removed trigger call elaboration score, as we simply wanted to model mean response call elaboration scores rather than elaboration changes relative to trigger calls.

#### AI declaration

AI was used as a search tool (Microsoft Copilot, Consensus), and for generating code templates as starting points for figures (Microsoft Copilot). The authors subsequently reviewed and edited the content as necessary and take full responsibility for the publication's final content.

## RESULTS

Unless otherwise stated, predicted probabilities presented here for focal predictors of interest are the estimated marginal means, with non-focal continuous covariates held at mean values and predictions averaged across all levels of non-focal categorical covariates (via ‘sjPlot’ R package: https://CRAN.R-project.org/package=sjPlot). We used an α of 0.05 to determine statistical significance.

### Response delay and response period curves

**Fig. 3.**
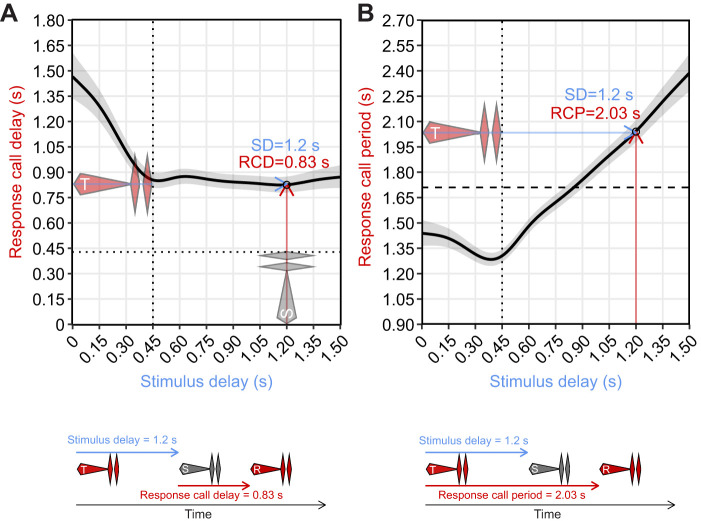
**Response delay and response period curves.** Top: results from our GAMM models (estimated marginal means and 95% confidence intervals; *n*=4249 responses from 39 males) with an arbitrary point on this curve highlighted for illustrative purposes (open circle). Bottom: visualization of the variables/values represented by this arbitrary point, to aid readers' understanding. (A) Response_Delay_GAMM results: average response delays to isolated low OP stimulus calls encountered at various stimulus delays. The red call approximates a typical trigger call (Ryan and Rand, 2003), with stimulus delays to the left of the vertical dotted line representing stimuli that likely overlapped trigger calls to varied degrees. The gray call represents the stimulus call, with the horizontal dotted line representing this call's duration. The middle section of the blue arrow extending through the trigger call to the open circle has been removed because of extensive overlap with the response curve, but can be inferred. (B) Response_Period_GAMM results: average response periods arising as a result of response delays shown in A. The horizontal dashed line at 1.71 s depicts the expected call period (ECP: median call period observed during dyadic chorusing: Larter and Ryan, 2024a). Thus, when the curve runs below this line, this indicates a shortening of call periods relative to the ECP, and vice versa. Intercepts of such curves (e.g. phase response curves) are typically equivalent to the ECP (see examples in Larter and Ryan, 2025). Conversely, here, the intercept is below the ECP. This likely arose because we did not systematically present stimulus calls to males at these short, overlapping, stimulus delays (<0.45 s). Rather, we obtained these data opportunistically when males called spontaneously shortly before onsets of stimulus calls presented at the longest delays. Our maximum delay was 1.5 s, meaning datapoints to the left of the vertical dotted line (in both plots) are strongly biased towards a subset of males that commonly exhibited much shorter call periods than average (<1.5 s; plotted intercept is just under 1.5 s). See Fig. S3 for a demonstration of this sampling bias. RCD, response call delay; SD, stimulus delay.

This species' response delay curve (Response_Delay_GAMM) is roughly piece-wise linear. It begins with a near-linear decrease in response delays to stimulus calls played at short stimulus delays that overlapped male trigger calls (0–0.45 s) (but see Fig. 3 caption for caveats). Then, it flattens out for all later, non-overlapping, stimulus delays (Fig. 3A). Though males exhibited variation in overall response curve shape (Fig. S2), this general shape predominated, and most variation was in curve intercepts. Other variables significantly influenced response delays (Table 1). Stimulus call ID influenced response delays {predicted response delay (s) [95% confidence interval (CI)]: low OP, 0.86 [0.81, 0.9]; intermediate OP, 0.94 [0.89, 0.99]; high OP, 0.77 [0.74, 0.81]}, higher trigger call elaboration scores decreased response delays slightly {response delay (s) −1 s.d., 0.87 [0.83, 0.91]; mean, 0.85 [0.81, 0.89]; +1 s.d., 0.84 [0.8, 0.88]}, and larger males exhibited shorter response delays {response delay (s) 1.3 g body mass, 0.91 [0.85, 98]; 1.7 g, 0.85 [0.81, 0.89]; 2.1 g, 0.79 [0.73, 0.85]}. The effects of water temperature {26.5°C, response delay (s) 0.86 [0.77, 96]; 27.5°C, 0.85 [0.81, 0.89]; 28.5°C, 0.85 [0.77, 0.93]}, and call onset time within trials {5 min, response delay (s) 0.86 [0.82, 9]; 17.5 min, 0.85 [0.81, 0.89]; 30 min, 0.85 [0.8, 0.89]} were negligible and non-significant. Results for Response_Period_GAMM were equivalent, just in the slightly different context of response periods rather than response delays; the flat latter arm of the response delay curve (Response_Delay_GAMM) yielded a response period curve (Response_Period_GAMM) that increased linearly in a 1:1 manner for all stimulus delays beyond those that overlapped trigger calls (Fig. 3B).

**Table 1.** Results of Response_Delay_GAMM (response delay∼stimulus delay) (*R*^2^=0.62)

Predictor variables	Estimate	CI	*P*	edf	Ref. d.f.	*F*	*P*
Linear terms							
(Intercept)	1.48	0.12–18.27	0.76				
Response call onset time	1	1.00–1.00	0.29				
Trigger call elaboration score	0.98	0.98–0.99	**<0.001**				
Stimulus call ID (intermediate OP)	1.1	1.07–1.13	**<0.001**				
Stimulus call ID (high OP)	0.9	0.88–0.93	**<0.001**				
Body weight	0.83	0.71–0.97	**0.02**				
Water temperature	0.99	0.91–1.09	0.89				
Smooth terms							
Smooth term (stimulus delay)				8.6	8.94	51.59	**<0.001**
Random intercept (male ID)				31.96	36	162.12	**<0.001**
Smooth term (stimulus delay, by male ID)				92.22	386	32.3	**<0.001**
Random Slope (response call onset time, by male ID)				29.69	38	51.51	**<0.001**
Random Slope (trigger call elaboration score, by male ID)				20.54	38	1.9	**<0.001**
Random slope (stimulus call ID, by male ID)				63.68	112	3.34	**<0.001**

Results for Response_Period_GAMM were similar (see Table S1). Predictor variables for GAMMs were not standardized prior to inclusion, with the exception of trigger call elaboration score, which was standardized within males. CI, confidence interval; edf, effective degrees of freedom; Ref. d.f., reference degrees of freedom; OP, overlap probability. Bold indicates significance.

### Effects of preceding motifs on responses to stimulus calls

**Fig. 4.**
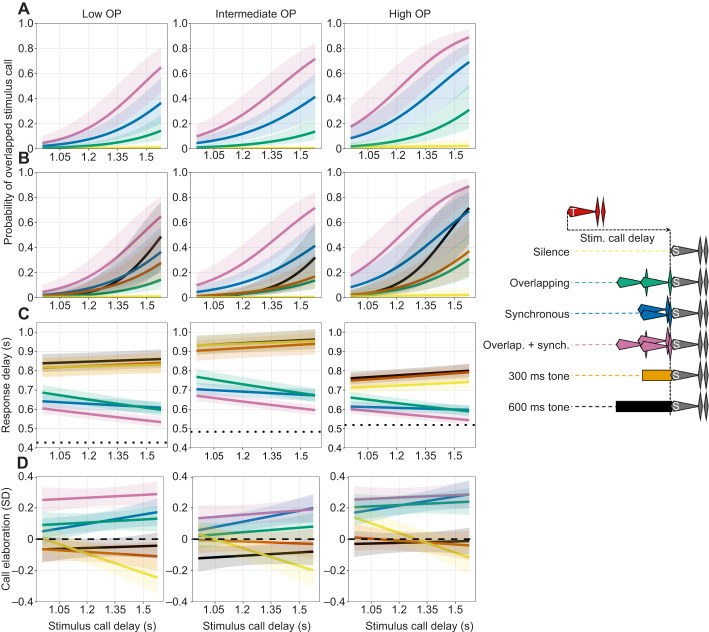
**Effects of preceding motifs on responses to stimulus calls.** Estimated marginal means and 95% confidence intervals from our mixed effects models, showing how the interaction between stimulus delay and acoustic motifs preceding stimulus calls influences various response call properties (for each figure, *n*=12,153 responses from 39 males). Curve colors correspond to colors of motif depictions on the right. (A) Predicted probabilities that response calls overlapped stimulus calls (Overlap_GLMM), shown only for silence and conspecific call motifs, to reduce clutter. (B) Predicted probabilities that response calls overlapped stimulus calls (Overlap_GLMM), shown for all motifs. (C) Predicted response delays for response calls that did not overlap stimulus calls (Response_Delay_LMM)*.* Dotted lines depict stimulus call durations. (D) Predicted call elaboration scores (standardized within males) relative to males' mean elaboration scores (Within_Male_Call_Elaboration_LMM); dashed line at 0 indicates within-male mean elaboration score.

For our mixed-effects models, model summaries are presented in Tables S2–S6, marginal effects of our main interactions of interest are visualized in Fig. 4, and marginal effects of control variables are presented in Table 2. See Table S7 for random effects summaries. *P*-values were calculated via likelihood ratio tests using the ‘afex’ package (https://CRAN.R-project.org/package=afex).

**Table 2.** Predictions and 95% CI for control covariates from our models, across a representative range of predictor values

Model name:	Overlap_GLMM	Response_Delay_LMM	Within_Male_Call_Elaboration_ LMM	Between_Male_Call_Elaboration_ LMM
Response variable:	Overlap probability	Response delay (s)	Response call elaboration within-males (s.d.)	Response call elaboration between-males (score)
Body mass	1.3 g: 0.33 [0.15, 0.59]	1.3 g: 0.58 [0.54, 0.61]	–	1.3 g: 2.4 [2.05, 2.82]
	1.7 g: 0.7 [0.53, 0.83]	1.7 g: 0.56 [0.54, 0.59]	–	1.7 g: 2.65 [2.41, 2.92]
	2.1 g: 0.92 [0.79, 0.97]	2.1 g: 0.55 [0.51, 0.59]	–	2.1 g: 2.93 [2.47, 3.47]
Water temperature	26.5°C: 0.39 [0.12, 0.76]	26.5°C: 0.59 [0.54, 0.64]	–	26.5°C: 2.53 [1.98, 3.22]
	27.5°C: 0.66 [0.48, 0.81]	27.5°C: 0.57 [0.54, 0.59]	–	27.5°C: 2.63 [2.39, 2.9]
	28.5°C: 0.86 [0.6, 0.96]	28.5°C: 0.55 [0.5, 0.59]	–	28.5°C: 2.74 [2.22, 3.37]
Response call onset time	5 min: 0.75 [0.59, 0.86]	5 min: 0.57 [0.54, 0.49]	5 min: 0.16 [0.07, 0.25]	5 min: 2.52 [2.28, 2.8]
	17.5 min: 0.68 [0.5, 0.82]	17.5 min: 0.57 [0.54, 0.49]	17.5 min: 0.25 [0.2, 0.31]	17.5 min: 2.65 [2.41, 2.91]
	30 min: 0.61 [0.41, 0.78]	30 min: 0.56 [0.54, 0.49]	30 min: 0.35 [0.26, 0.45]	30 min: 2.78 [2.5, 3.09]
Trigger call elaboration	−1 s.d.: 0.65 [0.46, 0.8]	−1 s.d.: 0.57 [0.55, 0.6]	−1 s.d.: −0.34 [-0.4, -0.28]	–
	Mean: 0.69 [0.51, 0.82]	Mean: 0.57 [0.54, 0.59]	Mean: 0.29 [0.23, 0.35]	–
	+1 s.d.: 0.72 [0.55, 0.85]	+1 s.d.: 0.56 [0.54, 0.58]	+1 s.d.: 0.92 [0.86, 0.98]	–

Predictions are for responses to stimulus calls preceded by the overlapping+synchronous motif at a stimulus delay of 1.5 s (i.e. highly arousing conditions), and predictions are averaged across all stimulus call IDs. Grayed cells highlight statistically significant relationships; see Tables S2–S6 for *P*-values.

#### Overlap_GLMM

Overlap probability was influenced by significant interactions between preceding motif and stimulus delay (*P*<0.001), and preceding motif and stimulus call ID (*P*<0.001), though the interaction between stimulus delay and stimulus call ID was non-significant (*P*=0.13). All stimulus call IDs were essentially never overlapped at any stimulus delay when preceded by silence (predicted overlap probabilities [95% CI] when preceded by silence at 1.5 s stimulus delay: low OP, 0.01 [0.0, 0.03]; intermediate OP, 0.01 [0.0, 0.02]; high OP, 0.02 [0.01, 0.07]). However, overlap probabilities increased when stimulus calls were preceded by our acoustic motifs, with the overlapping+synchronous motif inducing the highest probability of overlap (Fig. 4). Additionally, overlap probabilities increased, and differences among motifs widened, as stimulus calls were also presented at later stimulus delays. In contexts promoting overlap, the same hierarchy of stimulus call overlap probabilities originally observed in a live chorusing context was reproduced (predicted overlap probabilities [95% CI] when stimulus calls preceded by overlapping+synchronous motif at 1.5 s stimulus delay: low OP, 0.54 [0.35, 0.72]; intermediate OP, 0.63 [0.46, 0.78]; high OP, 0.84 [0.68, 0.92]). The 1000 Hz tone motifs induced similar overlap probabilities to certain motifs composed of conspecific calls, suggesting generic inhibition by acoustic stimulation is an important driver of overlapping responses. Higher body mass and higher trigger call elaboration scores significantly increased overlap probabilities, whereas males became significantly less likely to overlap stimulus calls as trials wore on (Table 2). Though our fixed effects explained a good proportion of variation [marginal *R*^2^ (proportion of variance explained by fixed effects)=0.3], most was explained by our random effects [conditional *R*^2^ (proportion of variance explained by fixed+random effects)=0.78]. The random intercept showed the highest variance (8.89), suggesting males differ widely in their baseline propensities to overlap rivals' calls.

**Fig. 5.**
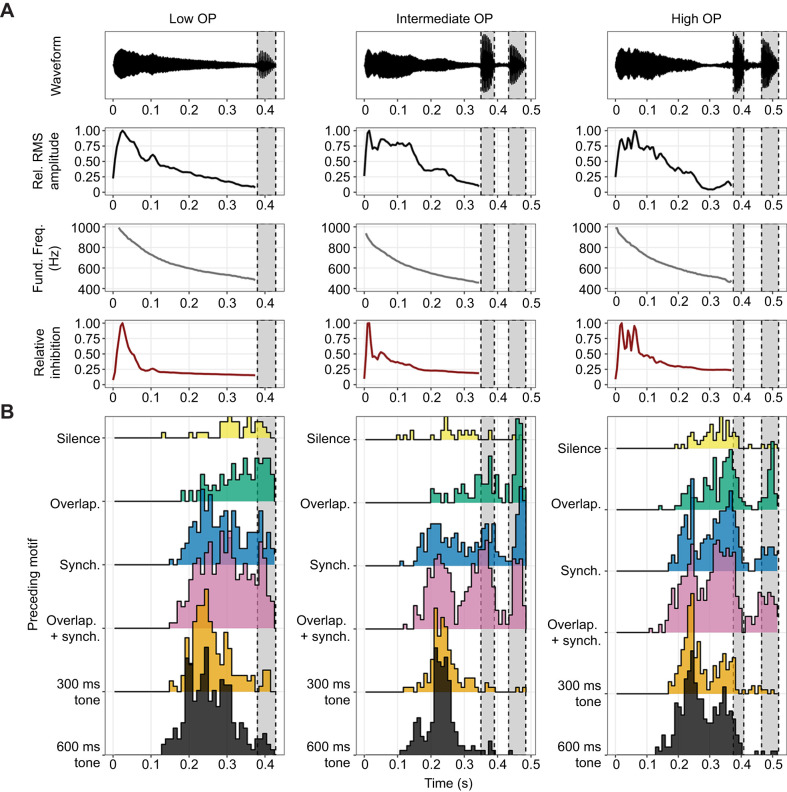
**Stimulus call properties and response delay histograms.** (A) Waveforms, relative root mean square (RMS) amplitude trajectory, fundamental frequency trajectory and relative inhibition trajectories by stimulus call ID. Relative inhibition trajectories were obtained by projecting simultaneous amplitude and fundamental frequency trajectories into a male sensory system model generated in a calling context (Larter and Ryan, 2024b; illustrated in fig. 2 of Larter and Ryan, 2025; see Supplementary Materials and Methods, ‘Supplemental Information H: Methodology for calculating Inhibition Trajectories for Stimulus Calls’, for details). (B) Ridgeline histograms (‘ggridges’ R package: https://CRAN.R-project.org/package=ggridges) showing when onsets of overlapping response calls occurred throughout stimulus calls when preceded by different motifs (10 ms bins). Many calls occurred during the latter parts of whines as has been discussed previously (Larter and Ryan, 2024a,c), but large, compact, peaks during secondary chucks are evident for intermediate and high OP calls; these calls were presumably triggered by offsets of initial chucks. Multiple peaks are also evident in the latter parts of whines of intermediate and high OP calls, and seem to arise as a result of inter-male variation in the timing of overlapping calls (see Fig. S5 for visualizations). See Fig. S6 for similar histograms for all responses (overlapping and non-overlapping).

Durations of our different stimulus calls correlated positively with their probabilities of being overlapped (Overlap_GLMM). This gave us pause, as our working theory was that overlap is driven primarily by frequency and amplitude patterns throughout calls (Larter and Ryan, 2024b,c), rather than generic features such as duration. However, a detailed look at overlap patterns revealed that the importance of duration arises indirectly as a result of increased call durations stemming primarily from additional chuck notes. Onsets of overlapping response calls occurred at several peaks during stimulus calls – most often during the latter portions of whines in accordance with our theory, but also tightly clustered during secondary chuck notes of multi-chuck stimulus calls (see intermediate OP and high OP in Fig. 5). This primarily occurred when multi-chuck stimulus calls were preceded by motifs composed of conspecific calls which induced the shortest response delays (Fig. 4, discussed below), rather than motifs composed of tones which induced long response delays. This suggests that this latter peak is generated by short-latency calls being triggered by the offsets of the initial chuck of multi-chuck stimulus calls. Thus, initial chucks may commonly trigger calls, but it is only when coupled with stimulation patterns that sufficiently shorten response delays that this results in this form of overlap.

To disentangle this form of overlap from the stereotyped overlap arising when species-typical whine amplitude and frequency trajectories trigger response calls (Larter and Ryan, 2024b), post-hoc we ran an identical model to Overlap_GLMM. Except, here, we only considered response calls to be overlapping stimulus calls if their onsets occurred prior to the ends of the initial chucks of intermediate OP and high OP calls (Restricted_Overlap_GLMM). Results differed somewhat from Overlap_GLMM, with intermediate OP having a slightly lower overlap probability than low OP (though these were essentially equivalent), but with high OP still having the highest overlap probability overall {predicted overlap probabilities [95% CI] when stimulus calls preceded by overlapping+synchronous motif at 1.5 s stimulus delay: low OP, 0.53 [0.33, 0.72]; intermediate OP, 0.49 [0.31, 0.67]; high OP, 0.7 [0.49, 0.85]}. See Fig. S4 for visualizations.

#### Response_Delay_LMM

For responses that did not overlap stimulus calls, response delays were influenced by significant interactions between preceding motif and stimulus delay (*P*<0.001), and preceding motif and stimulus call ID (*P*<0.001), though the interaction between stimulus delay and stimulus call ID was non-significant (*P*=0.18). The effects of motifs separated into two clear clusters (Fig. 4); stimulus calls preceded by silence and 1000 Hz tone motifs induced similarly long response delays, which increased slightly as stimulus calls were encountered at greater stimulus delays. Conversely, motifs composed of conspecific calls induced shorter response delays that tended to decrease somewhat as stimulus calls were encountered at greater stimulus delays, and response delay distributions (see Fig. S6) exhibited pronounced peaks ∼60 ms after the offsets of stimulus calls (near the lower reaction-time limits for this species: Larter and Ryan, 2024b). Stimulus call ID influenced average response delays in ways not in accordance with the hierarchy of stimulus call durations (low OP duration<intermediate OP<high OP) {predicted response delays (s) [95% CI] averaged across all preceding motifs at 1.5 s stimulus delay: low OP, 0.7 [0.67, 0.74]; intermediate OP, 0.79 [0.75, 0.83]; high OP, 0.67 [0.65, 0.7]}. This suggests that stimulus calls themselves aroused males to different degrees. Our control variables had only negligible and/or non-significant effects (Table 2). Here, most variation was explained by our fixed effects (marginal *R*^2^=0.39, conditional *R*^2^=0.73).

#### Within_Male_Call_Elaboration_LMM

Response call elaboration score, standardized to reflect within-male changes relative to typical call elaboration levels, was influenced by significant interactions between preceding motif and stimulus delay (*P*<0.001), and preceding motif and stimulus call ID (*P*=0.004), though the interaction between stimulus delay and stimulus call ID was non-significant (*P*=0.4). Here again, the same two clusters are evident as for Response_Delay_LMM (Fig. 4). Response calls following motifs composed of silence or 1000 Hz tones had elaboration scores that were similar to, or slightly less than, the trigger call that preceded them. Response calls following silence motifs also showed pronounced temporal effects, with longer stimulus delays inducing greater reductions in response call elaboration scores. Conversely, response calls following motifs composed of conspecific calls had elaboration scores higher than the trigger calls that preceded them, and this effect increased somewhat at greater stimulus delays. However, response call elaboration score was almost entirely predicted by the elaboration score of the trigger call immediately preceding it; marginal *R*^2^ for the full model was 0.47 (conditional *R*^2^=0.52), and removing trigger call elaboration score as a predictor reduced the marginal *R*^2^ to 0.04, whereas removing preceding motif only reduced it to 0.45. This demonstrates the inertia over time in call elaboration levels seen previously (Bernal et al., 2009).

#### Between_Male_Call_Elaboration_LMM

In agreement with previous work (Bernal et al., 2007; Larter and Ryan, 2024a), between-male differences in call elaboration scores were not significantly influenced by male body mass or water temperature, though they were positively associated (Table 2; see Fig. S7 for call elaboration score distributions). In fact, almost all variation in this model was explained by the random effects (marginal *R*^2^=0.06; conditional *R*^2^=0.76). However, recall that our call elaboration score scaled summed chuck amplitudes relative to the amplitude of the associated whine (Fig. 2 caption). Larger males in this species have louder whines on average (James et al., 2021), meaning that similar call elaboration scores in smaller and larger males likely translate to absolutely louder chucks in the latter.

## DISCUSSION

We investigated how conspecific stimulation patterns interacted with endogenous responsiveness rhythms to guide socially mediated response flexibility in túngara frogs. Our results revealed that response flexibility is driven on a call-by-call basis by a multifaceted cue to social context. This cue incorporates both the character of short-term conspecific stimulation experienced prior to each call, and how the timing of this stimulation interacts with responsiveness changes unfolding throughout call cycles. Overall, our results demonstrate that seemingly inactive periods within behavioral rhythms provide repeated assessment windows for fine tuning upcoming responses, and that these rhythms themselves afford males a source of temporal information regarding local social dynamics.

### Temporal responses to isolated conspecific calls

Phase-response curves (PRCs) have been characterized extensively in chorusing insects (Greenfield and Roizen, 1993; Sismondo, 1990; Walker, 1969). These can be complex and discontinuous functions, with males of some species exhibiting varied temporal responses to rivals' calls encountered at different points throughout their call cycles. Fewer frog PRCs are available, but those that are show simpler continuous functions that arise from males producing consistent short-latency responses to rival's calls encountered at all delays beyond the refractory period (Lemon and Struger, 1980; Loftus-Hills, 1974; Larter and Ryan, 2025). Túngara frogs exhibit this same pattern, with our response delay curve remaining flat for all stimulus delays beyond the initial refractory period, and with the response period curve showing a corresponding 1:1 linear increase (Fig. 3).

Though we did not systematically sample stimulus delays that overlapped male trigger calls (<0.45 s), opportunistic data from a subset of males suggest that stimulus calls at these delays cannot elicit the short-latency responses seen at later delays (illustrating the refractory period: Narins, 1982). However, the decreasing left arm of our curves suggests that stimulus calls encountered during the refractory period still shorten the current call period somewhat, and do so to greater degrees at later stimulus delays (also seen in *Pseudacris crucifer*: Lemon and Struger, 1980). This suggests that these overlapping stimulus calls are perceived to some degree and arouse males such that the next endogenously triggered call occurs sooner than it otherwise would have. The increasing effect at later delays likely occurs because larger portions of later overlapping stimulus calls are perceivable protruding beyond the ends of males' own trigger calls.

### Temporal responses in the context of conspecific stimulation patterns

Dyadic interactions represent but one of countless social contexts in which call-timing mechanisms must function, yet this is typically the sole context in which they are investigated. However, the simple and consistent response patterns exhibited by túngara frogs in a dyadic context (Fig. 3) belie the true versatility of their call-timing mechanism. This versatility was revealed only when we presented stimulus calls to males preceded by acoustic motifs that mimicked conspecific stimulation patterns typically encountered in different social environments (we discuss the effects of tonal motifs below). Isolated stimulus calls were essentially never overlapped at any stimulus delay. However, when stimulus calls were preceded by motifs consisting of combinations of overlapping and synchronous conspecific calls, males altered their call-timing responses; they increased their probabilities of overlapping stimulus calls in the stereotyped fashion seen in this species, and decreased the latencies of their response calls (Fig. 4). This was most pronounced for the motif featuring both overlapping and synchronous conspecific calls (overlapping+synchronous), which exhibited both the greatest duration and highest peak and average amplitude of the conspecific motifs used here. Furthermore, we observed a salient interaction between motifs and males' endogenous responsiveness rhythms in influencing overlap probabilities and response latencies, with the escalatory effects of conspecific motifs increasing synergistically as stimulus calls were presented to males at later delays (Fig. 4).

Shifts between alternating interactions and stereotyped overlap in this species seem to arise from males altering how permissive their gap-detection mechanisms are regarding the magnitude of a release from inhibition that is sufficient to trigger a call (Larter and Ryan, 2025). Increased overlap prevalence in larger choruses seems to arise as male gap-detection mechanisms become increasingly permissive within these social contexts, thus making the moderate release from inhibition shortly following rivals' whine onsets an increasingly salient call trigger (Larter and Ryan, 2024b). Our results here suggest that males increase the permissiveness of their gap-detection mechanisms as they experience increasingly intense conspecific stimulation between calls, and as this stimulation extends ever later throughout their call cycles. As choruses grow larger, males of this species increase call amplitude (Larter et al., 2023) and call elaboration (Bernal et al., 2007; Larter and Ryan, 2024a), chorus duty cycles increase (Larter and Ryan, 2024a), and additive amplitude spikes arising from overlapping and synchronous calls become increasingly prevalent (Larter and Ryan, 2024a). Thus, the intensity and duty cycle of stimulation received from rivals increases predictably as choruses grow, making it an informative emergent cue to a male's current social context that can be used to guide adaptive shifts in interaction tactics.

This use of emergent social cues to fine-tune interaction patterns is even more impressive when considering the time scales over which this occurs. We played all stimulus call/stimulus delay/motif combinations to males in a random order within the same playback trial, with each stimulus presentation interspersed with a standardized alternation interaction to reduce carry-over effects. Thus, the varied effects that different motif and delay combinations had on male responses suggests that call-timing mechanism parameters are altered anew each call cycle in response to stimulation patterns sampled immediately prior to each impending call. This allows calling strategies that are highly responsive to unpredictable short-term fluctuations in collective chorusing dynamics. These results demonstrate that inactive periods during behavioral cycles afford valuable repeated opportunities to sample dynamic social scenes, such that each impending response can be tailored to its immediate collective behavioral context (Ariel et al., 2014).

Once these call-timing mechanism parameters have been altered by inter-call stimulation patterns, the responses that ultimately terminate each call cycle arise as this mechanism then interacts with the acoustic properties of rivals' calls encountered subsequently. Hence the varied responses to different stimulus call IDs observed here when they were preceded by the same motifs at the same delays. When using the same overlap metric (Overlap_GLMM), we experimentally recovered the same hierarchy of overlap probabilities for our stimulus calls as was observed in a live six-male chorus (Larter and Ryan, 2024c). This provides strong support that our experiment appropriately replicated the key mechanistic drivers of overlapping responses at play in real choruses.

### Túngara frog call-timing mechanisms in their ecological context

Our emerging picture of túngara frog call-timing mechanisms reveals properties that will be advantageous across the varied range of social environments in which males can find themselves. Females of this species prefer non-overlapping calls (Legett et al., 2019), so males alternate their calls without overlap in smaller (≤3 males) choruses in which it is possible to do so (Larter and Ryan, 2024a). However, overlap becomes increasingly unavoidable in larger choruses (Larter and Ryan, 2024a). Thus, to maintain the high call rates that females also prefer (Ryan and Keddy-Hector, 1988), males become increasingly tolerant of overlapping rivals' calls when emergent stimulation patterns suggest they are in a social environment in which overlap is unavoidable. Moreover, the sensory tuning of their gap-detection mechanism strongly prioritizes overlap of the stereotyped form described (Larter and Ryan, 2024b). This form of overlap imposes lower attractiveness costs than any other form; thus, attractiveness costs of obligatory overlap are duly minimized (Larter and Ryan, 2024a).

In fact, as response patterns are updated on a call-by-call basis, the tradeoff between calling at high rates and calling at times of reduced interference can be continually optimized in light of short-term fluctuations in temporally heterogenous chorus noise (Larter and Ryan, 2025). Males can immediately become permissive of overlapping rivals' calls in this minimally costly way when intense inter-call stimulation extending late into their call cycle indicates that encountering silent gaps within the time horizon of the current call period is unlikely. Yet, they can immediately switch back to prioritizing calling within silent gaps again when sparser inter-call stimulation patterns suggest lengthy silences may be forthcoming. Indeed, overlap and alternation both occur in larger choruses (Larter and Ryan, 2024a). Similarly, intense inter-call stimulation also reduced response delays, allowing males to most effectively capitalize on short-lived gaps in ongoing chorus noise when they occur (Larter and Ryan, 2024a).

### Call elaboration in the context of conspecific stimulation patterns

In túngara frogs, males increase call elaboration in more competitive choruses (Bernal et al., 2007; Larter and Ryan, 2024a). In agreement with this, here call elaboration was increased somewhat by conspecific motifs typical of larger choruses. We observed the same general motif arousal hierarchy as was seen in temporal contexts (overlapping+synchronous motifs being the most arousing), and similar (though less pronounced) increases in this effect as stimulus calls were encountered at later delays. Conversely, when motifs were preceded by silence, we saw a tendency to reduce call elaboration, with this reduction becoming starker as stimulus calls were encountered at later delays (yellow lines in Fig. 4D). Túngara frog calling strategies have been influenced by the behavior of predatory bats and parasitic flies that eavesdrop on male calls to locate them as prey, and show similar preferences for complex calls to females (Bernal et al., 2006; Tuttle and Ryan, 1981). Consequently, males rapidly cease calling when their nearby rivals cease, as this suggests these rivals may have detected an incoming eavesdropper (Dapper et al., 2011). The apparent rapid decay of arousal over the course of call cycles exhibiting extended silences is likely related to this defensive response.

Overall though, response call elaboration was primarily driven by the elaboration of the trigger call preceding it. Thus, though overlap probability and response latencies were primarily responsive to short-term inter-call stimulation patterns, call elaboration is subject to a high degree of inertia over longer stretches of calling (Bernal et al., 2009) and is only influenced slightly by shorter-term stimulation patterns. This suggests that call elaboration and temporal aspects of responses are controlled by different neural circuits that integrate sensory data over different time horizons. However, that motifs did influence response call elaboration somewhat, and that males calling more elaborately exhibited slightly higher overlap probabilities (Table 2) suggests these circuits partially intersect and interact.

### Similar and divergent influences of conspecific and tonal motifs

Though conspecific motifs had broadly analogous escalatory effects on all dimensions of calling responses, the effects of tonal motifs showed interesting differences across response properties. Tonal motifs induced longer response delays and slight reductions in call elaboration, thus having similar effects to silence for these call properties (Fig. 4). However, tonal motifs had a similarly positive effect to overlapping and synchronous conspecific call motifs on overlap probabilities. This suggests that, though conspecific stimulation influences all of these response properties in similarly escalatory ways, it may be different aspects of this stimulation that are salient. For instance, stimulation by more specific conspecific patterns, such as species-typical amplitude and frequency trajectories (Ryan, 1983; Walkowiak, 1988), may be necessary to induce shorter response delays and increased call elaboration. Conversely, more generic effects of conspecific stimulation patterns, such as inhibition by high-amplitude acoustic stimulation more generally, may be the salient feature driving overlap probabilities; hence our tonal motifs having similar effects.

It may be that broader tuning of the elements of call-timing mechanisms that calibrate gap-detection behavior to background interference levels is beneficial, as this would allow callers to be similarly responsive to other forms of biotic and abiotic noise which can also interfere with call transmission (Vélez et al., 2013). Whereas, more specificity in the tuning of elements dealing with, for example, call elaboration would ensure males only incur the risks/costs of calling elaborately when conspecific competition is high and elaboration will be beneficial, and eavesdropper risks are diluted by calling rivals (Bernal et al., 2006; Ryan et al., 1981; Tuttle and Ryan, 1981). Whatever the reason, we see that different properties of conspecific stimulation influence different dimensions of a single response behavior (Walkowiak, 1988). This emphasizes that responses by individuals within collectives arise from numerous parallel perceptual processes interacting in complex ways with the dynamic multi-dimensional sensory scenes generated by their group-mates (Hein, 2022; Williams et al., 2023).

### Conclusions

Our findings demonstrate that túngara frogs sample emergent patterns in conspecific scenes during the inactive periods preceding each call, and then alter their interaction mechanisms to promote beneficial responses given these revealed social dynamics. Furthermore, salient features of conspecific scenes for guiding these adjustments are multifaceted, involving both the character of stimulation perceived and when it is perceived relative to males' endogenous responsiveness rhythms. Pieces of this picture are evident in other collective behavioral contexts, suggesting these results have broader relevance beyond chorusing. For instance, behavioral adjustments in response to broader emergent properties of conspecific sensory scenes facilitate collective hunting in spiders (Chiara et al., 2022) and collective locomotion in zebrafish (Harpaz et al., 2021). Additionally, alternating phases of sampling collective dynamics, and responding in light of these dynamics, underpin group cohesion during collective locomotion in locust and zebrafish groups (Ariel et al., 2014; Harpaz et al., 2017). Thus, cues arising as emergent fluctuations in conspecific sensory scenes are filtered through individual responsiveness rhythms likely have widespread importance for guiding appropriate interaction patterns within dynamic behavioral collectives.

## Supplementary Material

10.1242/jexbio.250982_sup1 Supplementary information
